# Method and Device of All-in-Focus Imaging with Overexposure Suppression in an Irregular Pipe

**DOI:** 10.3390/s22197634

**Published:** 2022-10-09

**Authors:** Shuangjie Wang, Qiang Xing, Haili Xu, Guyue Lu, Jiajia Wang

**Affiliations:** School of Mechanical Engineering, Nantong University, Nantong 226019, China

**Keywords:** all-in-focus, high reflection, irregular pipe, free-form surface, non-Lambertian surface, overexposure, image fusion

## Abstract

To avoid depth-of-field mismatches caused by the changes in pipe structure and image overexposures caused by highly reflective surfaces while radial imaging irregular pipes, this paper proposes a novel all-in-focus, adaptable, and low scene-coupling method that suppresses overexposures in support of fault detection. Firstly, the pipeline’s radial depth distribution data are obtained by sensors, and an optimal all-in-focus imaging scheme is established by combining camera parameters. Secondly, using digital imaging technology, the high reflection effect produced by disparate light sources is comprehensively evaluated for overexposure suppression. Thirdly, a device is designed for imaging non-Lambertian free-form surface scenes under low illumination, providing the sequence images needed for the next step. Lastly, specific digital fusions are made to the sequential images to obtain an all-in-focus final image without overexposure. An image-quality analysis method is then used to measure the efficacy of the system in obtaining the characteristic information of the inner surfaces of an irregular pipe. Results of the experiment show that the method and device used are able to distinguish small 0.5 mm wide lines ranging from 40–878 mm depth and are capable of providing efficient image support for defect inspection of irregular pipes and free-form surfaces amongst other irregular surfaces.

## 1. Introduction

Irregular pipes (e.g., those with S-shaped inlets) have special profile and variable cross-sectional features [1,2] characterized by continuous and irregular changes in the axial and radial directions, and the special non-Lambertian coating sprayed on its inner surface shows high reflectivity [3]. Thus, it is difficult to acquire the high-quality images needed for rapid digital fault detection.

Among the contemporary techniques, axial and radial imaging are the most common. Axial imaging refers to single-viewpoint methods (e.g., closed-circuit television [4], fisheye [5], single-reflection [6,7], and catadioptric panoramic annular lens [8,9]), in which the imaging device is placed along the axial direction of the inner pipeline. This results in a circular ring image whose effects directly depend on the structural regularity and coincidence degrees between the visual and pipeline axes, resulting in an image distortion-correction algorithm needing to be applied [6,10]. Any sudden changes in pipeline appearance or deviations along the visual axis can reduce imaging effects and detection accuracy. Thus, it is difficult to directly apply axial methods to irregular pipes. Radial imaging implies that the visual axis of the imaging device is perpendicular to the inner surface of the pipeline, from which information is obtained directly [11]. Although this method can be adapted to circular pipes of varying sizes by changing lenses, irregular pipes typically provide frequent and large radial depth-of-field (DoF) changes that make information acquisition prohibitively difficult and time-consuming. Imaging systems with expanded DoFs are helpful, and researchers have offered several related solutions (e.g., wavefront coding [12,13], special flat lensing [14], and light-field imaging [15,16]) that improve DoF handling at the expense of spatial resolution. However, these results are unsuitable for reliable defect detection. To achieve the all-in-focus imaging requirements needed for irregular pipes, more recent studies have applied multifocus fusion methods using sequence images [17,18]. These methods have good fusion effects that can be applied to a wide range of tasks, but they rely on passive focusing algorithms that are also unsuitable for irregular pipes, particularly due to the lack of texture gleaned in the low illumination [19,20]. Later, Liu [21] proposed another method of depth-image segmentation, which achieved good all-in-focus results, but the graph-based algorithm was unsuitable for continuous imaging. Notably, when imaging surfaces have large depth changes, it tends to be difficult to control the segmentation depth. Thus, a multicamera fusion scheme with a multifocal lens [22] can help resolve the large DoF problem. However, the required equipment is bulky, the solution is overly complex, and the system does not work well inside irregular and small pipelines requiring large depth spans.

Active illumination is often required when imaging pipe interiors. However, the highly reflective and freely changing structural features quickly lead to overexposures, which reduce the utility of the acquired information for fault detection. To mitigate these negative effects, Shao [23] devised a digital micro-mirror device (DMD) to realize pixel-level spatiotemporal modulations of illumination, the principle of which is that the reflected light (adaptive stripes with spatiotemporal modulation) modulated by DMD is projected through a triangular prism, mirror, and projection lens onto a workpiece with a highly reflective surface, and a uniform illumination is formed on the workpiece surface to highlight the defect information. Liu [24] introduced a study that uses a coaxial light source to obtain the first coaxial optical images to identify the bearing center coordinates and text information; then, under the multiangle light, images are obtained at different angles in turn, and all the defects of the bearing can be highlighted after image fusion. Feng [25] realized the acquisition of surface defect information of small highly reflective parts through high dynamic imaging technology and improved the quality of acquired images. Chen [26] designed a dual lighting system with a large area and high-brightness illumination from both sides of the steel ball; this design improved the light uniformity on the steel ball surface, thereby avoiding the generation of light spots and creating good conditions for subsequent detection. Although these methods addressed specific highly reflective objects and achieved decent results, most are meant to be used with the outer surfaces of objects. Notably, when imaging devices and algorithms are designed for specific tough jobs, their performance tends to be tightly coupled to the unique configuration; thus, they poorly adapt to changing scenes.

In summary, to obtain a method for solving the DoF mismatch problem while supporting overexposure suppression imaging of the insides of irregular pipes, this paper first proposes a cross-modal all-in-focus imaging device and strategy based on the target surface depth using a lighting and imaging method of overexposure suppression that depends on high-reflection prior information. Then, an imaging device is introduced, and an imaging experiment of 0.5 mm wide fine lines is carried out on the highly reflective surface with a maximum depth of 878 mm. Subsequently, an image fusion is carried out and the best all-in-focus image with no overexposure is obtained. The proposed method and device can provide efficient image support for defect inspection of special-shaped pipes, free-form surfaces, and other unconventional scenes, and it has good practical ap-plication value. 

The structure of the paper is organized as follows: firstly, the background and key issues of all-in-focus imaging and imaging on high reflect surface are presented in Section 1, along with an overview of the relevant literature. Section 2 elaborates the method of all-in-focus imaging through fusing the depth date of imaging surface, as well as the method for overexposure suppression on surface which has high reflection character. In Section 3, an imaging device, which has a high-resolution camera, depth sensors, light sensors, and four evenly spaced light sources, is designed, and three imaging experiments and evaluations of the imaging effects are applied to verify the effectiveness and superiority of the above proposed method. Lastly, Section 4 summarizes the key technologies and achievements of this paper.

## 2. Materials and Methods

### 2.1. Cross-Modal All-in-Focus Imaging Strategy

**Figure 1:**
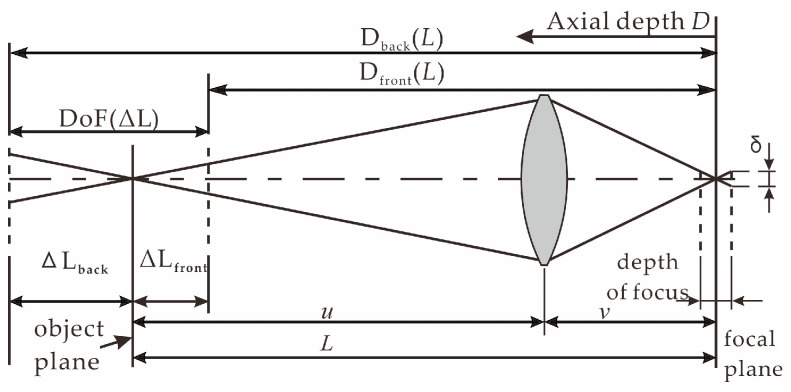
Degree-of-freedom principal diagram.

The DoF is the key imaging feature of a pinhole camera, as shown in Figure 1, and it is determined by the diameter of the allowable circle of confusion, *δ*, focal length, *f,* and lens *F*-value, which can be characterized by the front and back DoFs:

(1) $$\left\{ \begin{array}{l} \Delta L_{\mathrm{front}}=\frac{F\times \delta \times L^{2}}{f^{2}+F\times \delta \times L}\\ \Delta L_{\mathrm{back}}=\frac{F\times \delta \times L^{2}}{f^{2}-F\times \delta \times L} \end{array}\right.,$$

where ΔL_front_ represents the front DoF, ΔL_back_ represents the back DoF, *L* represents the shooting distance from the focal plane to the photosensitive element, and *δ* is a hyperparameter set according to the sharpness requirement. Generally, the photosensitive elements size is selected in [1, n] pixels for the *δ* number. In this study, to clearly demonstrate the law of front and back scenes along an object plane of distance *L*, we let

(2) $$\left\{ \begin{array}{l} D_{\mathrm{front}}(L)=L-\Delta L_{\mathrm{front}}=L-\frac{F\times \delta \times L^{2}}{f^{2}+F\times \delta \times L}\\ D_{\mathrm{back}}\left(L\right)=L+\Delta L_{\mathrm{back}}=L+\frac{F\times \delta \times L^{2}}{f^{2}-F\times \delta \times L} \end{array}\right.,$$

where D_front_(*L*) is the depth function of the DoF’s front edge (Figure 1), and D_back_(*L*) is the depth function of its back edge. According to Equation (3)’s Gaussian imaging formula, for a fixed-focus pinhole imaging system, the image distance, *v*, can be changed by moving the lens focus. Thus, changes in *v* allow objects to be clearly imaged in the corresponding DoF under different object distances, *u*.

(3) $$\frac{1}{f}=\frac{1}{u}+\frac{1}{v}.$$

**Figure 2:**
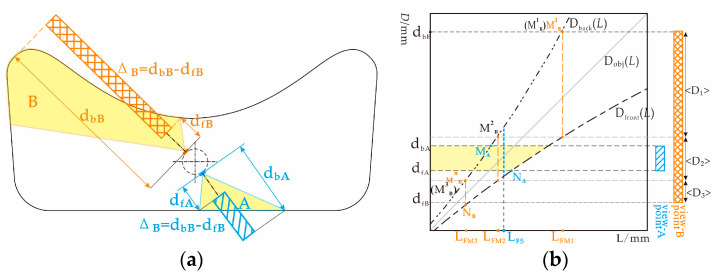
DoF principle of pinhole and cross-modal all-in-focus imaging strategy: (**a**) radial section diagram of the irregular pipe; (**b**) adaptive hybrid focusing principle.

The distance from the inner surface of the irregular pipe to the geometric centroid of the respective radial cross-sections is large. When the camera is at the center of the cross-section from different viewpoints, the large DoF differences required by each viewpoint cause single- or multifocus imaging techniques to fail to match the appropriate DoF. To capture images in such scenes, a DoF strategy of pinhole and cross-mode all-in-focus imaging is needed, as shown in Figure 2. 

As shown in Figure 2a, d_fX_ and d_bX_ respectively represent the depths of the nearest and farthest points of the imaging object from the *X*(*X* = A, B) viewpoint. The depth span, Δ*_X_*(Δ*_X_ =* d_b_*_X_ −* d_f_*_X_*), is the total DoF required for Viewpoint X. The curves of the D_front_(*L*) and D_back_(*L*) functions are shown in Figure 2b. Let the depths, D = d_f_*_X_*, of the closest point of the target and D = D_front_(*L*) intersect at point N*_X_*(D_front_^−1^(d_f_*_X_*), d_f_*_X_*). The depths, D = d_b_*_X_*, of the farthest points of the target and D = D_back_(*L*) intersect at point M*_X_*(D_b_^−1^(d_b_*_X_*), d_b_*_X_*). The object plane depth function, D_obj_(*L*) = *L*. When D_front_^−1^(d_f_*_X_*) ≥ D_back_^−1^(d_b_*_X_*) for any L_FS_∈(D_back_^−1^(d_b*X*_), D_front_^−1^(d_f*X*_)), there is (d_f_*_X_*, d_b_*_X_*) ∩ (D_front_(L_FS_), D_back_(L_FS_)) where the current depth range is included in any DoF with L_FS_ as the object-plane depth, including the camera imaging range at Viewpoint A. To ensure good imaging quality, the intermediate depth of the scene can be used to focus the image in the single-focus mode, as follows:

(4) $$D_{\mathrm{Fsingle}}(d_{f}{}_{A},d_{\mathrm{bA}})=\frac{D_{\mathrm{front}}{}^{-1}\left(d_{\mathrm{fA}}\right)+D_{b}{{}_{\mathrm{ack}}}^{-1}\left(d_{\mathrm{bA}}\right)}{2}{\;D}_{f}{}^{-1}\left(d_{\mathrm{fA}}\right)\geq D_{b}{}^{-1}\left(d_{\mathrm{bA}}\right),$$

where D_Fsingle_(d_fA_, d_bA_) denotes the depth of the object plane when the depth range is (d_fA_, d_bA_). Correspondingly, when the camera is at Viewpoint B for any L_FM_ ∈ (D_back_^−1^(d_bB_), D_front_^−1^(d_fB_)), there are (d_fB_, d_bB_) ⊄ (D_front_(L_FM_), D_back_(L_FM_)), such that when D_front_^−1^(d_fB_) < D_back_^−1^(d_bB_), the DoF corresponding to any object plane, L_FM_, cannot completely cover the current surface, and multiple segmented focusing images are required. The mathematical relationship of the corresponding conditions is expressed as

(5) $$\left\{ \begin{array}{l} \left(<D_{1}>\cup <D_{2}>\cup \cdot \cdot \cdot \cup <D_{n}>\right)\cap \left(d_{\mathrm{fB}},d_{\mathrm{bB}}\right)=\left(d_{\mathrm{fB}},d_{\mathrm{bB}}\right)\\ n=N_{\min} \end{array}\right.,$$

where <D_n_> is the depth range of each segment after the target depth is segmented, and N is the number of segmentations, where N_min_ is the minimum value. The object plane depth, D_obj_(L_FM_*_j_*)*,* corresponding to depth range <D*_j_*>, is analyzed using the abscissa of point M*^j^*_B_, where *j* = 1, 2, …, n, and n = 3 in Figure 2b. Then, for Viewpoint B, when D_front_^−1^(d_fB_) < D_back_^−1^(d_bB_), all sub-focal plane positions in the multifocus mode can be obtained. {D_Fmulti_(d_fB_, d_bB_)} = {D_FM1_, D_FM2_, ⋯⋯, D_FM_*_j_*}, and

(6) $$D_{\mathrm{FM}j}=\left\{ \begin{array}{l} D_{\mathrm{back}}{}^{-1}\left(d_{\mathrm{bB}}\right),(j=1)\\ D_{\mathrm{back}}{}^{-1}\left(D_{\mathrm{front}}\left(D_{\mathrm{FM}\left(j-1\right)}\right)\right){,(D}_{f}\left(D_{\mathrm{FM}\left(j-1\right)}\right){>d}_{\mathrm{fB}}) \end{array}\right.,$$

where D_FM*j*_ represents the depth of each object plane corresponding to <D*_j_*> of multifocus imaging, and D_FM*j*_ = D_obj_(L_F__M*j*_) = L_F__M*j*_. In this paper, the process of dividing (*d_f_*, *d_b_*) into {<D_1_>, <D_2_>, …, <D_n_>} is called “depth segmentation”. The process of calculating a {<D_1_>, <D_2_>, …, <D_n_>} that matches {D_FM1_, D_FM2_, …, D_FMn_} is called “DoF matching”. Accordingly, in view of the large radial depth span of an irregular pipe, a cross-modal adaptive all-in-focus imaging strategy is proposed, as shown in Equation (7).

(7) $$\left\{D_{F}\left(d_{f},d_{b}\right)\right\}=\left\{ \begin{array}{l} \left\{D_{\mathrm{Fsingle}}(d_{f},d_{b})\right\}{\;,D}_{\mathrm{front}}{}^{-1}\left(d_{f}\right)\geq D_{\mathrm{back}}{}^{-1}\left(d_{b}\right)\\ \left\{D_{\mathrm{Fmulti}}(d_{f},d_{b})\right\}{\;,D}_{\mathrm{front}}{}^{-1}\left(d_{f}\right)<D_{\mathrm{back}}{}^{-1}\left(d_{b}\right) \end{array}\right.,$$

where {D_F_(*d_f_*, *d_b_*)} is the set of depth positions of the object plane after solving the (*d_f_*, *d_b_*) depth interval. By imaging the object planes in the set, the all-in-focus sequence images in the current field of view can be obtained.

### 2.2. Lighting and Imaging Strategy of Overexposure Suppression

**Figure 3:**
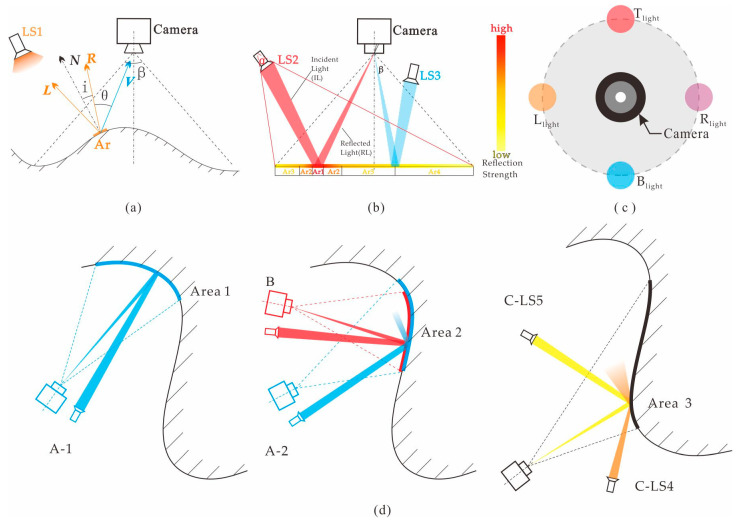
Non-Lambertian free surface lighting effect: (**a**) reflection diagram of phone model; (**b**) characteristic of high reflection at different light sources LS2 and LS3 on the plane surface; (**c**) schematic diagram of lighting device; (**d**) the influence of the positional relationship between camera, light source and surface on the reflection performance.

The classic Phong model [27] can be used to quantitatively describe the relationship between the amount of light observed as a function of the surface profile and light and viewing angle. As shown in Figure 3a, ***L***, ***N***, ***R***, and ***V*** are the incident light, imaging surface normal, reflected light, and observation vectors, respectively. When the small-area diffuse light source (LS) is independent and unique, Equation (8) is obtained as follows:

(8) $$I=k_{d}\times I_{\mathrm{pd}}\times \cos i+k_{s}\times I_{\mathrm{ps}}\times \cos^{m}\theta \left(0^{\circ }\leq \theta \leq 90^{\circ }\right),$$

where k_d_ × I_pd_ × cos*i* and k_s_ × I_ps_ × cos*^m^θ* are the diffuse and specular reflection components, respectively, and *m* is the reflection light convergence index related to surface smoothness. As the observation angle, *θ*, decreases, there is a region named area-of-reflect (Ar), in which the specular reflection component increases sharply by the *m-*th power, which is a high-reflection region.

When a small area diffuse light source is used for direct illumination, the effect of the specular reflection component of the non-Lambertian plane is shown in Figure 3b. Under the action of LS1, Ar1, Ar2, Ar3, and Ar4 are the direct high-reflection, high-reflection transition, conventional reflection, and low-reflection areas, respectively. Consequently, the specular reflection intensity decreases. According to Equation (8) and Figure 3b, the high-reflection areas created by light sources of different paths do not completely overlap. Therefore, under a fixed viewing angle, the high-reflection areas, Ar1 and Ar2, can only be generated by light source LS2 in the incident optical path, IL–RL; however, this is not true for other light sources, such as LS3. Therefore, it is possible to obtain image sequences with highly reflective position differences by separately illuminating and imaging with small light sources at different positions to recover surface information through image fusion [26,28]. 

As shown in Figure 3d, A-1, A-2, and B are imaging systems with illuminations in which the positional relationships between the camera and the small light source are relatively fixed. C-LS1 and C-LS2 are imaging systems with illuminations that lack fixed positional relationships between the camera and light source. The reflection problem of free-form surfaces mainly results from the coupling of three factors: the surface feature structure of the free-form surface, camera pose, and light-source pose. The reflection type is set by the bright (high reflection) or dark (non-high reflection) field-forward caused by these three conditions [29]. See Figure 3d for poses A-1 and A-2 of the same viewpoint position and A-1, or A-2 and B, facing the same area but with different poses. Even under the same camera pose, C, when lighting conditions LS4 and LS5 differ, the imaging effects are not highly reflective at C-LS4, but are highly reflective at C-LS5, and different surface and lighting conditions can produce the same effects. Under freely changing imaging conditions and the mutual influence of reflective factors, the system must adopt different lighting schemes to adapt to the various changes.

Because the surfaces and camera poses are separately determined for each image, we designed a dynamic lighting device comprising four independently controllable small diffuse light sources in the same plane. The device produces low coupling effects between the imaging characteristics and surface features. As shown in Figure 3c, each light source is combined using the same intermediate camera to create a relatively fixed position, and a lighting and imaging overexposure suppression matching strategy is proposed as follows:

- Four light sources are used to separately provide illumination and pre-imaging.
- The reflection of the image formed under the illumination of each light source is calculated to obtain each source’s prior information.
- The prior information that produces the least high reflection is chosen.

If high reflections cannot be avoided in this fashion, the complementary information between images formed by two light sources is used to suppress the high reflection effect via fusion. The decision conditions are determined as follows:

- Condition-Ca: If a single light source, *a* or *b*(*a*, *b*∈{T_light_, B_light_, L_light_, R_light_}), can avoid the high-reflection overexposure, it is selected for supplementary light,
  (9) $$\mathrm{Size}\left({\mathrm{IMG}}_{a}\right)=0,\;\mathrm{StdDev}\left({\mathrm{IMG}}_{a}\right)=\min\left(\mathrm{StdDev}({\mathrm{IMG}}_{b})\right).$$
- Condition-Cb: If highly reflective overexposure cannot be avoided, a combined lighting scheme with dual light sources, *a* and *b*(*a*, *b*∈{T_light_, B_light_, L_light_, R_light_}), is selected for supplementary illumination, and the decision equation is expressed as

(10) $$\mathrm{LC}\left({\mathrm{IMG}}_{a},{\mathrm{IMG}}_{b}\right)=\max\left(k_{O}\times \mathrm{ORI}\left({\mathrm{IMG}}_{a},{\mathrm{IMG}}_{b}\right)+k_{M}\times \mathrm{SOI}\left({\mathrm{IMG}}_{a},{\mathrm{IMG}}_{b}\right)\right),$$

where IMG*_a_* represents the image obtained when light source *a* provides illumination. Size(IMG*_a_*) indicates the size of the area of the overexposure in IMG*_a_*, T_light_, B_light_, L_light_, and R_light_ represent the light sources at the location of top, bottom, left, and right, respectively, and StdDev(IMG*_a_*) represents the overall standard deviation of image IMG*_a_*.

In Equation (10), LC(IMG*_a_*,IMG*_b_*) is the decision value of the light-source combination (LC), and the overall reflective intensity (ORI *=* 2 − 2/(1+ e^(−k^^α ×^ ^α)^)) represents the overall highly reflective imaging condition under two light sources. The spot overlap intensity (SOI = 3/(3 + 10 × e^(kβ × (β − 1))^) is used to characterize the overlap between the image spots formed under the two light sources, where α and β are the normalized and coincident spot areas after the AND operation (S_and_(IMG*_a_*,IMG*_b_*)). The OR operation (S_or_(IMG*_a_*,IMG*_b_*)) pertains to spots α = S_and_(IMG*_a_*,IMG*_b_*)/S_img_ and β = S_or_(IMG*_a_*,IMG*_b_*)/S_img_, and k_O_ and k_M_ are the ORI and SOI weights, respectively, in the decision-making system, where k_O_ + k_M_ = 1. kα and kβ represent the total and coincident spot area penalty coefficients, respectively. A larger penalty coefficient results in a stronger attenuation effect of the increase in α and *β* on the overall decision-making result. A larger LC value of the decision result of the decision-making formula leads to a better final imaging effect. The important function of this formula is the selection of the two light sources that provide the smallest total spot area for illumination and imaging under the condition that the spot overlap is the smallest possible. The sequence of images obtained by this decision are obtained after fusion to produce the image with the best high-reflection suppression from the current viewpoint.

### 2.3. Imaging Control and Fusion Scheme

**Figure 4:**
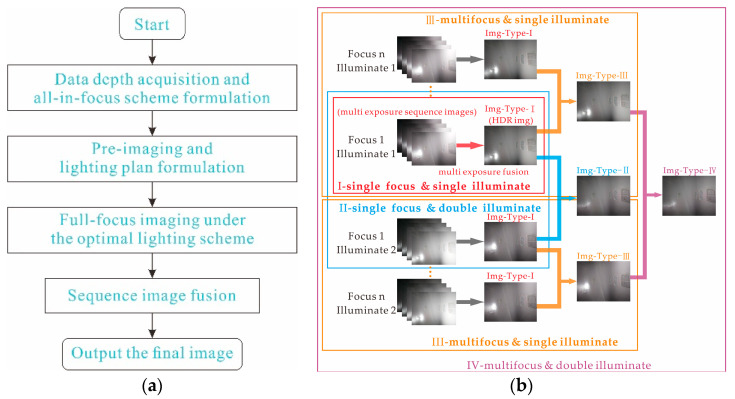
Imaging and fusion process: (**a**) the process of image acquisition by the imaging method in this paper; (**b**) the method and process of image fusion in this paper. I, the process in the red box indicates that the sequence images obtained under the imaging combination I in step 3 are subjected to multi-exposure fusion to obtain the Img-Type-1; the processes in the blue box and the orange box in II and III respectively indicate that, under imaging combinations II and III, by using wavelet fusion or multifocus fusion, the images of Img-Type-2 or Img-Type-3 can be obtained; the process shown in the purple box IV shows that image of Img-Type-4 can be obtained by fusing two images of Img-Type-3 under combination IV by multifocus fusion.

Owing to the complex relationships among surface features, illumination angles, and camera poses, sample quality is often significantly reduced. To remedy this problem, the adaptability of the imaging device is improved, as shown in Figure 4a, using the following adaptive decision-making control strategy:

1. Depth data acquisition and all-in-focus scheme: Using the depth sensor to obtain the depth distribution of the unknown curved surface in the current field of view, depth data are applied to Equation (7).
2. Pre-imaging and lighting plan: Using the depth data, different light sources are used for single-exposure pre-imaging at a fixed photosensitive level at the middle depth of the current surface and analyzing quality by judging whether there is overexposure or calculating the exposure conditions. The acquired prior information of high reflectivity under each light source is used to form the lighting plan using Equations (9) and (10).
3. Full-focus imaging under an optimal lighting scheme: The combination of four imaging scenarios is present (i.e., I: single-focus single-illumination scene with small DoF; II: single-focus dual-illumination scene with small DoF; III: multifocus single-illumination scene with large DoF; and Ⅳ: multifocus dual-illumination scene with large DoF). Then, the all-in-focus scheme of Step 1 and the lighting scheme of Step 2 are used to match one of the four preset combinations, execute the illumination scheme once, and image each object plane separately.
4. Sequence image fusion: After acquiring the sequence images, a specific fusion scheme (e.g., multifocus or wavelet) is performed on the sequence images according to the combined method chosen in Step 3. The image fusion process in the four scenarios proceeds as described below.

As shown in Figure 4b, the output images of each mode are marked as Img-Type-I, Img-Type-II, Img-Type-III, and Img-Type-Ⅳ. During object plane imaging under each light source, multi-exposures of the four photosensitive levels are performed by changing the exposure time, and fusion is performed to obtain the high-dynamic range (HDR) image needed to solve the problem of insufficient dynamics and poor brightness uniformity under single imaging. This then facilitates the expression of internal defects and other information needed to obtain high-definition images in the full DoF. This process is indicated by the red arrow in Combination I in Figure 4b. 

## 3. Experimental Verification

### 3.1. All-in-Focus Imaging Device with Overexposure Suppression

**Figure 5:**
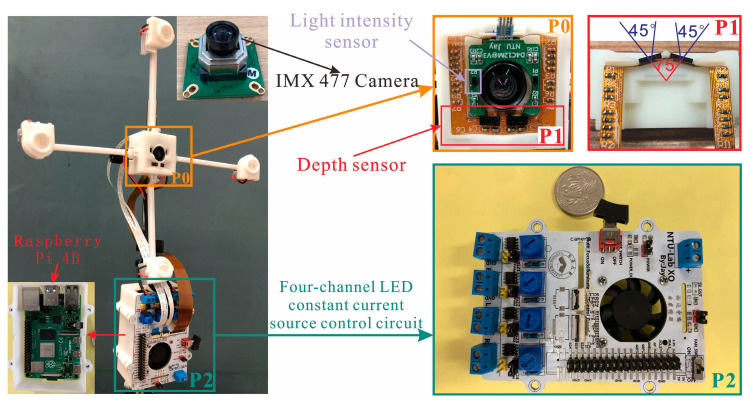
Composition of the imaging device.

Spatial resolution is an important performance parameter for measuring the ability of an imaging system to resolve fine defects. According to past research [30], the spatial resolution, R_space_, of radial imaging can be described as follows:

(11) $$R_{\mathrm{space}}=\frac{f}{l}=\frac{size_{pixel}}{size_{obj}},$$

where *f* is the focal length, *l* is the distance between lens and object, *l ≈ L*, *size_pixel_* is the single-pixel size of the occupied photosensitive element, and *size_obj_* is the minimum width of the defective object. To achieve the imaging requirements of *size_obj_* with 0.5 mm wide fine lines at *L* = 1000 mm in the irregular pipe, SONY IMX477 is selected as the photosensitive imaging element. According to Equation (12), the lens has a focal length, *f*, of 3.9 mm, aperture value, *F*, of 2.8, and field of view (FoV) of 75 × 52. For the depth sensor selection, the ultra-miniature multiarea depth sensor, VL53L5CX, with a resolution of 8 × 8 and an FoV of 45° × 45° was selected, and the two were combined as shown in Figure 5. Due to the excellent multizone ranging ability of the sensor, this device can measure the depth data of a plane, and its measurement accuracy reached 5% under the condition of this paper. A high-brightness light-emitting diode (LED) lamp bead with a color temperature of 5500 K was selected as the light source, and a polymethylmethacrylate diffuser lens with a diameter of 15 mm and an illumination angle of 120° were selected as the condensing element. The light source array, ultra-miniature area depth sensor, and geometric installation relationship with the camera are shown in Figure 5.

**Figure 6:**
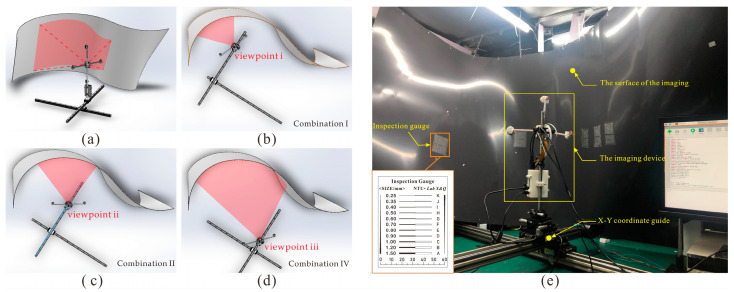
Experimental scene: (**a**) experimental scene model; (**b**) viewpoint i; (**c**) viewpoint ii; (**d**) viewpoint iii. When the model is at the location of viewpoints i, ii, and iii, the type of imaging combination becomes combinations I, II, and IV, respectively. (**e**) The actual scenario, where the clarity of the image can be judged by observing the thin line on the “inspection gauge”.

Controlling the exposure of sequential images is crucial to the accuracy of pre-imaging information and the acquisition of multiple-exposure sequential images. Exposure control is determined by the aperture value, International Standards Organization (ISO) sensitivity, and exposure time, and it is closely related to the stable light-field intensity. Because the overall reflection of incident light on the curved surface differs greatly per light source, an independent four-channel constant-current source-drive circuit and proportional–integral–derivative (PID) control algorithm are used to achieve the constant control of light intensity, as shown in Figure 6. The CN5711 constant-current LED driver, which has excellent current stability and brightness retention over a wide temperature range, is used to maintain light-level consistency between images. The algorithm uses light-intensity feedback to modulate the brightness of each source using pulse-width modulation. The PID control formula is shown in Equation (13), where *e* is the current error, and *e*_t−1_ is the last error originating from the feedback data of the two light intensity sensors (Figure 5). Simultaneously, to ensure the consistency of final sequence images, the process is controlled according to the consistency parameters presented in Table 1.

(12) $$C=K_{p}\times e_{t}+K_{i}\times \sum_{t=1}^{N}\times e_{t}+K_{d}\times \left(e_{t}-e_{t-1}\right).$$

### 3.2. Imaging Experiments

To verify the high-quality adaptability of the proposed imaging device under arbitrary DoFs, the experimental setup shown in Figure 6a was applied. A thin, gray, specular reflective iron sheet with diffuse reflection characteristics was used to build the scene (Figure 6e). The device was placed on a height-adjustable x–y coordinate slide to simulate different positional and attitudinal perspectives. The film ruler shown in the rectangle was attached to the curved surface and used to judge the final clarity. The device was designed to adaptively match the scene to combination Types I–IV based on the acquired depth and pre-imaging information. In this study, three viewpoints, *x*(*x* = i*,* ii*,* iii), of different combination types were imaged in the dark environment to verify the adaptability and efficacy of the proposed strategy and device. The viewpoint positions are shown in Figure 6b–d.

#### 3.2.1. Single-Focus and Illumination under Small DoF

**Figure 7:**
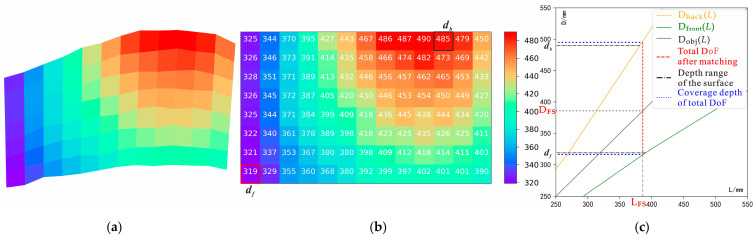
Decision-making process of the all-in-focus scheme at viewpoint i*:* (**a**) result of surface simulation at viewpoint *i*; (**b**) data of depth distribution at viewpoint i; (**c**) all-in-focus imaging scheme at viewpoint i.

The positional relationship between the camera and scene was adjusted to Viewpoint i, as shown in Figure 6b. According to the process of Figure 4a, an imaging experiment was performed using the developed imaging device. The experimental steps and results are as follows:

1. As shown in Figure 7b, by detecting the depth distribution of the current Viewpoint i to obtain the contour surface-depth data matrix, the maximum and minimum depths are *d_b_* = 485 mm and *d_f_ =* 319 mm, respectively, because D_front_^−1^(*d_f_*) ≥ D_back_^−1^(*d_b_*), according to the all-in-focus imaging strategy of Equation (5). If the current mode is single-focus, then the camera automatically sets the depth plane as D_FS_ = (D_front_^−1^(*d_f_*) + D_back_^−1^(*d_b_*))/2 = 386 mm for the final all-in-focus imaging scheme, as shown in Figure 7c.
2. After selecting the intermediate depth, D_mid_ = 402 mm, of the object plane, rapid pre-imaging under light sources of T_light_, B_light_, L_light_, and R_light_ are controlled according to the parameters in Table 1.

**Figure 8:**
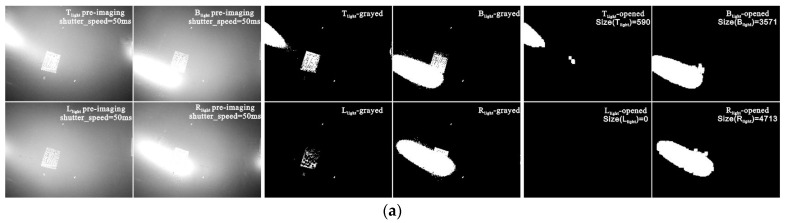

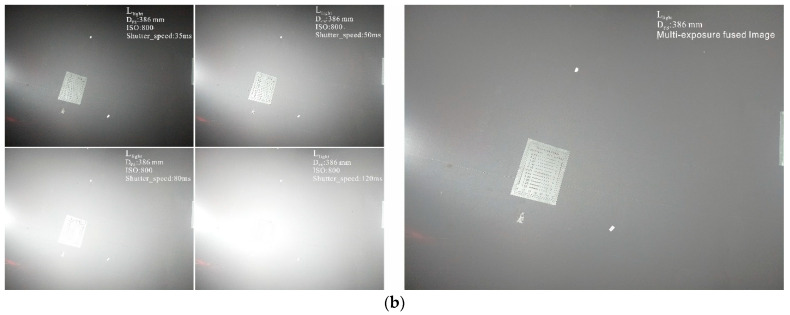
Decision-making process of lighting scheme and final image at viewpoint i: (**a**) decision-making process of lighting scheme; (**b**) HDR process of final image, where we fuse four images with different exposure levels, which are shown in the left part of the figure (**b**), into the right HDR image.

To improve the operation speed, the obtained image is reduced to 10% of the original for binarization where the threshold, *TH*, is 245. Then, a 5 × 5 opening operation is performed to obtain image with interference removed. The spot area Size(IMG*_a_*) is calculated for *a* ∈ {T_light_, B_light_, L_light_, R_light_}, and the optimal spot area under different light sources is discerned as shown in Table 2. The results show that L_light_ would not cause overexposure, which satisfies decision Condition Ca of Equation (9). Here, L_light_ is the final decision. Typical image-processing is illustrated in Figure 8a.

The final imaging scheme from Viewpoint i combines the full-focus scheme of Step 1 and the lighting scheme of Step 2, and D_FS_ = 386 mm is selected as the object plane for HDR imaging under L_light_. The imaging consistency control parameters are listed in Table 1.

As shown in Figure 8b, the multi-exposure fusion algorithm [31] is used on the sequence images to obtain the HDR image.

#### 3.2.2. Single-Focus and Dual Illumination under Small DoFs

**Figure 9:**
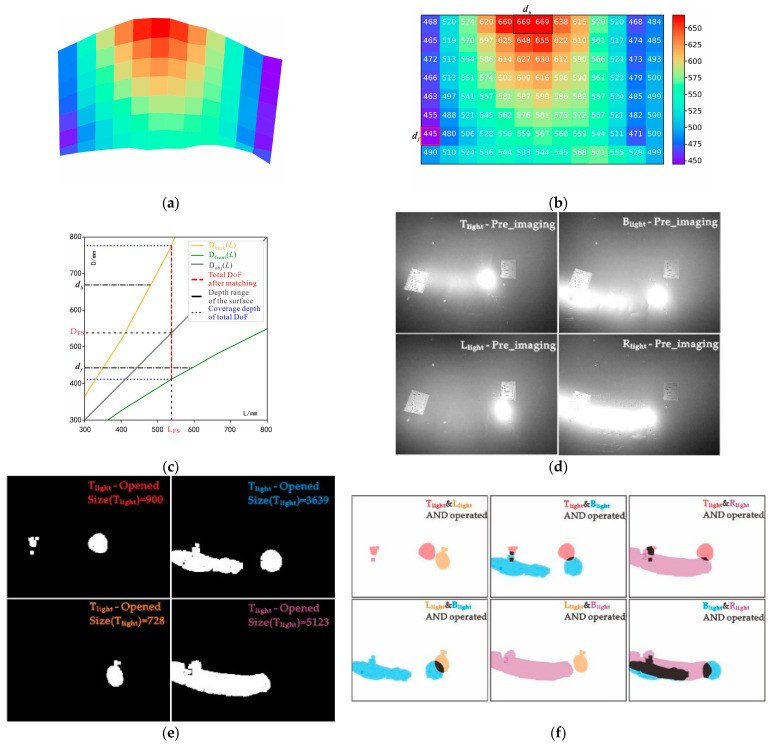
Decision-making process under viewpoint ii: (**a**) surface simulation; (**b**) data of depth distribution; (**c**) all-in-focus imaging scheme; (**d**) images of pre-imaging; (**e**) pre-imaging images after “open operation”, where the four different colors in this figure represent the different light sources. For example, the image with a red color character represents the image obtained under T_light_, and the blue, orange, and purple colors represent B_light_, L_light_, and R_light_ respectively. (**f**) Inferred images after “operation” between two different pre-imaging images, where the meaning of the color is similar to that shown in Figure 9e, and the area with black color represents the inferred area where the spots of different light sources coincide.

As shown in Viewpoint ii (Figure 6c), while single-focus imaging with a small DoF, if single-illumination Condition Ca cannot be satisfied, a dual light-source combined image is required. First, as with Viewpoint i, Step 1 is performed with the depth distribution data (Figure 9a–c), and the object plane depth, D_FS_ = 538 mm, is chosen to achieve the all-in-focusing scheme. 

Step 2 is performed next to obtain the pre-image single-illumination overexposure information from four light sources (Figure 9d,e). It can be seen that all single-light-source images suffer from overexposure. Hence, the ORI, SOI, and LC of the overexposed image (Table 3) are examined to determine the optimal combinations of two light sources under Condition Cb (Figure 9f). The black-filled area represents the overlapping overexposed area. A smaller area indicates a better final fusion effect. From the decision result, LC, in Table 4, the T_light_ and L_light_ combination obtains the best high reflection suppression effect.

Step 3 is then performed; a D_FS_ of 538 mm is chosen for the object plane, and multi-exposure sequence images are obtained under the separate illuminations of T_light_ and L_light_.

**Figure 10:**
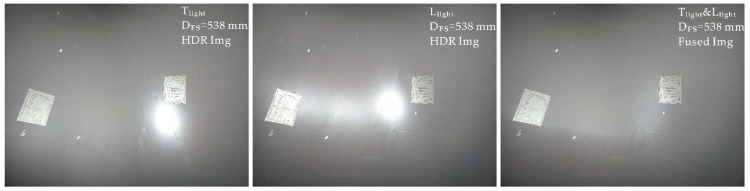
Overexposure suppression process under combined lighting.

Lastly, in Step 4, multi-exposure fusion is performed according to the Viewpoint i steps to obtain the optimal HDR images under two light sources. Due to the character smoothness of wavelet fusion method in image processing [32], wavelet-domain over-exposure fusion is then performed on the obtained HDR images while retaining low-level components (Figure 10).

#### 3.2.3. Multifocus and Dual Illumination under Large DoF

**Figure 11:**
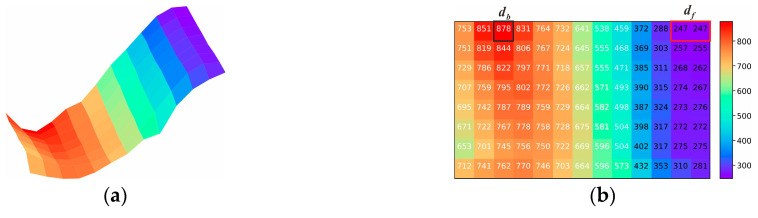

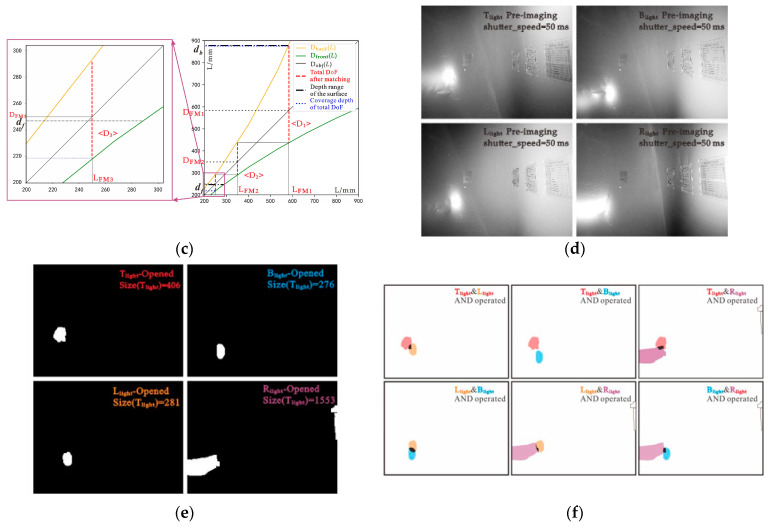
Decision-making process under viewpoint iii; (**a**) surface simulation; (**b**) data of depth distribution; (**c**) all-in-focus imaging scheme; (**d**) images of pre-imaging; (**e**) pre-imaging images after “open operation”, where the four different colors in this figure represent the different light sources. For example, the image with a red color character represents the image obtained under the T_light_, and the blue, orange, and purple colors represent B_light_, L_light_, and R_light_ respectively. (**f**) Inferred images after “operation”. Images are inferred between two different pre-imaging images, where the meaning of the color is similar to that shown in Figure 11e, and the area with black color represents the inferred area where the spots of different light sources coincide.

**Figure 12:**
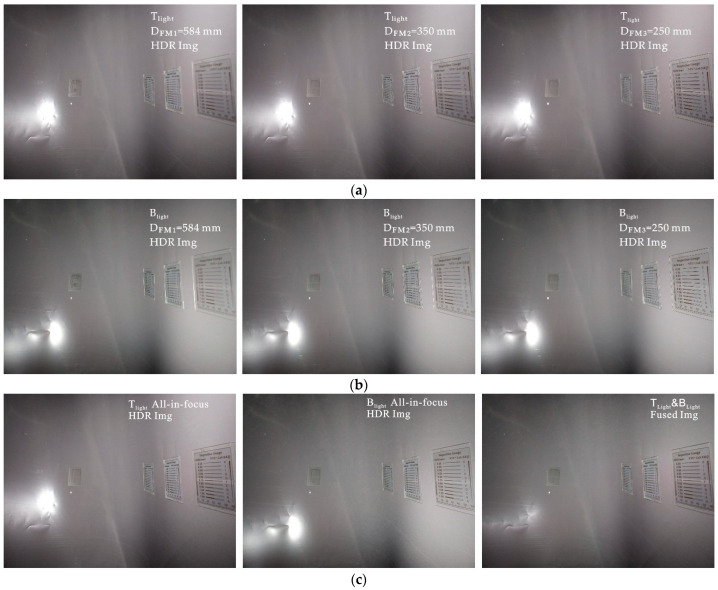
All-in-focus imaging with overexposure suppression under viewpoint iii: (**a**) three HDR images under T_light_ with three depths of object plane, D_FS1_ = 584 mm, D_FS2_ = 350 mm, and D_FS3_ = 250 mm; (**b**) three HDR images under B_light_ with three depths of object plane, D_FS1_ = 584 mm, D_FS2_ = 350 mm, D_FS3_ = 250 mm; (**c**) on the left is the all-in-focus HDR image which is obtained by fusing the three images under T_light_ in (**a**), in the middle is the all-in-focus HDR image which is obtained by fusing the three images under B_light_ in (**b**), and in the right is the the all-in-focus image without overexposure, which is obtained by fusing the left and the middle all-in-focus images.

The first two imaging experiments used small DoFs; however, for large cases, the problem of an insufficient DoF may be encountered. As shown in Figure 6d, Viewpoint iii has large depth multifocus features (Combination IV), including high reflectivity under Viewpoint ii. The efficacy of the proposed illumination imaging strategy under complex multifocus and combined illumination conditions is demonstrated using the Viewpoint iii experiment via the following steps:

1. According to Step 1, the ranges of the current depth as detected by the depth sensor were found to be *d_f_* = 247 and *d_b_* = 878. The depth matrix and surface diagram are shown in Figure 11a,b. As D_front_^−1^(*d_f_*) < D_back_^−1^(*d_b_*), the current focus mode is selected. Then, according to Equation (7), the depth distribution data are used to segment the current surface with depth-matching (Figure 11c). The assignment results for the object plane depth of the final all-in-focusing scheme are listed in Table 5. Unlike Viewpoints i and ii, Viewpoint iii requires depths of D_FM1_ = 584 mm, D_FM2_ = 350 mm, and D_FM3_ = 250 mm, as shown in Table 5.
2. Pre-imaging steps such as those in the previous experiments are performed to determine the lighting scheme. The pre-imaging and decision parameters are listed in Table 1 and Table 3, respectively, and the lighting decision-making process and data are shown in Figure 11d–f and Table 6, respectively. By comparing the LC results in Table 6, the combination of T_light_ and B_light_ are selected as the required combination to provide lighting.
3. As with Viewpoint ii, under T_light_ and B_light_, the multi-exposure image sequence at object plane depths of D_FM1_ = 584 mm, D_FM3_ = 350 mm, and D_FM2_ = 250 mm is acquired.
4. According to the fusion process shown in Figure 4b, the multi-exposure sequence images are first fused to obtain the HDR image according to the combination of each object plane’s depth and light-source condition (Figure 12a,b). For the multifocus HDR images obtained from the same light source, the method in [17] is used to perform multifocus fusion to obtain the all-in-focus HDR image under Viewpoint iii, and a wavelet fusion operation like the one from Viewpoint ii is used on the all-in-focused HDR images under light sources of T_light_ and B_light_ to suppress the high reflectivity and restore the information of the overexposed area. The final fusion results are shown in Figure 12c.

### 3.3. Effect Evaluation of All-in-Focus Imaging with Overexposure Suppression

The performance quality of the detailed imaging surface information at various depths was subjectively and objectively evaluated for the all-in-focus fusion of large DoFs and the information recovery effects after overexposure suppression.

#### 3.3.1. Evaluation of the All-in-Focus Imaging Effect

The post-all-in-focus fusion image should have better texture feature expression than any single image taken prior to fusion due to the improvements to global sharpness. We compared the results before and after fusion of the three focus images under Viewpoint iii with light source T_light_, as shown in Figure 12a, and we objectively and subjectively evaluated the all-in-focus effects.

##### Objective Evaluation of the All-in-Focus Effects

The objective evaluation calculated image sharpness on the basis of statistical image gradient and sharpness evaluations. The image gradient evaluation adopted the energy of gradient (EOG) and Tenengrad methods, and the Vollath function was chosen on the basis of the statistical results. Furthermore, the improvement in detail expression between the final image and each original image could be individually compared as a function of the signal-to-noise ratio (SNR), and peak signal-to-noise ratio (PSNR) [32].

➀ EOG function

The EOG function takes the square sum of the differences in gray value between a pixel and the adjacent pixels in the *x*- and *y*-directions as the gradient value of each, which are accumulated as input to the sharpness evaluation function. After averaging all pixels, the expression is

(13) $$V_{\mathrm{EOG}}=\frac{1}{X\times Y}\times \sum_{x}\sum_{y}\left\{{\left[g\left(x+1,y\right)-g\left(x,y\right)\right]}^{2}+{\left[g\left(x,y+1\right)-g\left(x,y\right)\right]}^{2}\right\}.$$

➁ Tenengrad function

The Tenengrad function uses the Sobel operator to calculate the gradient value representing the sharpness of the image edge in the horizontal and vertical directions. After averaging the gradient values of all pixels, the expression is

(14) $$V_{\mathrm{Tenengrad}}=\frac{1}{X\times Y}\times \sum_{x}\sum_{y}\left(G_{x}{}^{2}\left(x,y\right)+G_{y}{}^{2}\left(x,y\right)\right),$$

where Gx(*x*,*y*) and Gy(*x*,*y*) are the gradient values of the pixel in the horizontal and vertical directions at (*x*,*y*), respectively, and its formula is as follows:

(15) $$\left\{ \begin{array}{l} G_{x}\left(x,y\right)=g\left(x,y\right)⊗g_{x}\\ G_{y}\left(x,y\right)=g\left(x,y\right)⊗g_{y} \end{array}\right.,$$

where ⊗ is the convolution symbol, and g_x_ and g_y_ are the horizontal and vertical templates of the Sobel operator, which are defined as $g_{x}=\left[\begin{array}{l} -1\;0\;1\\ -2\;0\;2\\ -1\;0\;1 \end{array}\right],{\;g}_{x}=\left[\begin{array}{l} -1\;-2\;-1\\ \;\;\;0\;\;\;\;\;\;0\;\;\;\;\;\;0\\ \;\;\;1\;\;\;\;\;\;0\;\;\;\;\;\;1 \end{array}\right]$.

➂ Vollath function

The Vollath autocorrelation function reflects the similarity between two points in space. The edges of the clear texture details in an image are clear and sharp, and the correlation between the pixels is low, whereas the texture details in the out-of-focus area are blurred, and the correlation between image pixels is high. The calculation result reflects the similarity of all adjacent pixels, thus evaluating overall image quality. The Vollath function is expressed as follows:

(16) $$V_{\mathrm{Vollaths}}=\frac{1}{X\times Y}\times \sum_{x=1}^{X-2}\sum_{y=1}^{Y}\left(g\left(x,y\right)\times \left|g\left(x+1,y\right)-g\left(x+2,y\right)\right|\right).$$

➃ Signal-to-noise ratio

(17) $$\mathrm{SNR}=10\mathrm{lg}\frac{\sum_{x=0}^{X-1}\sum_{y=0}^{Y-1}p{\left(x,y\right)}^{2}}{\sum_{x=0}^{X-1}\sum_{y=0}^{Y-1}{\left[\left(p\left(x,y\right)-q\left(x,y\right)\right)\right]}^{2}}.$$

➄ Peak signal-to-noise ratio

(18) $$\mathrm{PSNR}=10\mathrm{lg}\left[\frac{255^{2}}{\mathrm{MSE}}\right],$$

where MSE is the mean square error,

(19) $$\mathrm{MSE}=\frac{1}{X\times Y}\sum_{x=0}^{X-1}\sum_{y=0}^{Y-1}{\left\|p\left(x,y\right)-q\left(x,y\right)\right\|}^{2}.$$

In the evaluation functions of Sections above, g(*x, y*) is the pixel value at (*x, y*) of the image in ➀–➂, p(*x, y*) is the pixel value at (x, y) of the original image in ➃–➄, q(*x, y*) is the pixel value at (*x, y*) of the fused image in ➃–➄, and X and Y are the numbers of rows and columns in the image pixel matrix, respectively. The calculation results are listed in Table 7 and Table 8.

The results of the objective evaluation show that, compared with the original images, those obtained from multifocus sequence fusion were significantly improved according to the sharpness quantification index, indicating that the overall sharpness of the fused images was significantly improved.

##### Subjective Evaluation of the Effects of All-in-Focus Fusion

Subjective evaluation was performed by directly observing the details and expressing the power of the images on the surface at each depth before and after all-in-focus imaging. Subsequently, the efficacy of the control strategy was ascertained.

**Figure 13:**
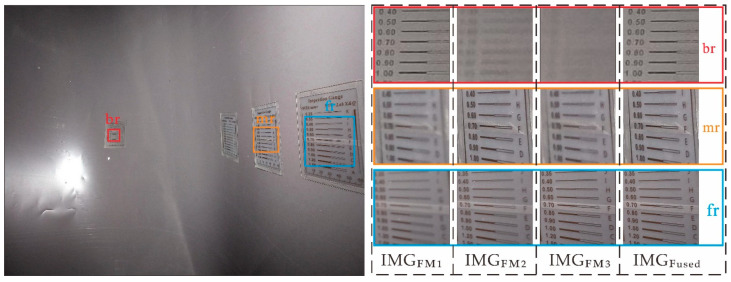
Comparison of results before and after all-in-focus.

As shown in Figure 13, there are three regions, back region (br), middle region (mr), and front region (fr), representing different depths of the surface. The details of the br, mr, and fr regions in the IMG_FM*j*_ (*j* = 1, 2, 3) at different object plane depths, D_FM*j*_ (*j* = 1, 2, 3), under Viewpoint iii were analyzed subjectively. Taking the horizontal comparison of the detailed image of area br in the red frame of Figure 13 as an example, image IMG_FM1_ had the most prominent detail expression at this depth, and the expressivities of IMG_FM2_ and IMG_FM3_ decreased with the decrease in object plane depth. The blurriness of image region br was positively correlated to the depth of the IMG_FM1_, IMG_FM2_, and IMG_FM3_ object planes. Meanwhile, the fused image, IMG_Fused_, had a similar detailed performance capability to IMG_FM1_, and depth regions mr and fr followed similar rules. By comprehensively comparing the clarity laws of br, mr, and fr, it can be found that, when facing the inner wall of the irregular pipe in the depth range of 247–878 mm from Viewpoint iii, compared with IMG_FM*j*_ after a single focus, which only represents the surface texture of a certain depth range, the fused image, IMG_Fused_, successfully distinguished surfaces at different depths with fine streaks and good performance, indicating that the imaging device and strategy effectively obtained pipeline surface information at various depths.

#### 3.3.2. Evaluation of Information Recovery Effect after Suppressing Overexposure

**Figure 14:**
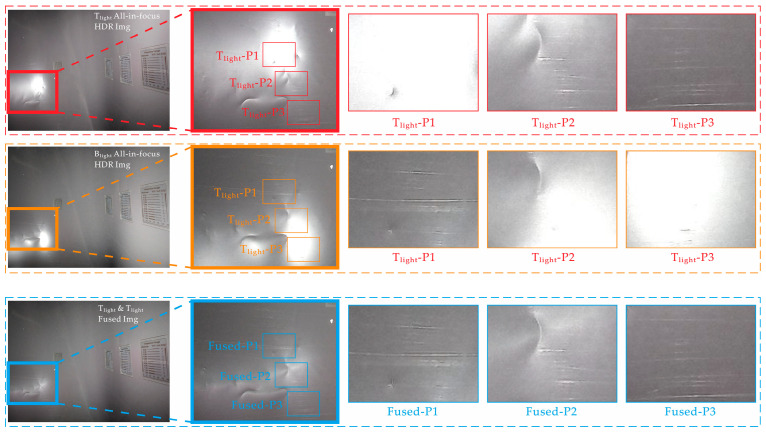
Comparisons of multifocus renderings.

To evaluate the efficacy of lighting and information recovery for the overexposed area, an example was provided via the selection judgment of the lighting source chosen under Viewpoint i, which showed that the proposed control strategy effectively selected the most suitable light source, thus ensuring lighting and imaging quality. However, when the overexposure problem cannot be overcome by changing the light-source positions, a sequence of images with non-overlapping overexposed areas can be generated using two light sources at different positions. As shown in Figure 14, detailed texture information of the overexposed area was recovered by fusion.

Figure 14 compares the details of the all-in-focus images under different light sources (T_light_ and B_light_) and after suppressing overexposure (Fused) in the same area and range. Considering the horizontal contrast between the detailed images under T_light_ as an example (outlined in red), T_light_-P1, T_light_-P2, and T_light_-P3 are the high-reflection overexposure and transition areas under the light source and the non-overexposed area, respectively. The overexposed area loses the ability to express texture details owing to pixel saturation. As the area transitions to a non-overexposed area, the detailed expression gradually increases. By longitudinally comparing the texture details of *X*_light_-P1, *X*_light_-P2, and *X*_light_-P3 (*X* = T_light_, B_light_, and Fused) areas, it can be seen that, after overexposure suppression, the overexposed spots were removed after magnifying the image details after suppression. Hence, it can be clearly seen that the texture information (e.g., scratches and dents) were recovered well, demonstrating that the device has a good overexposure suppression capability. These results show that overexposed spots in highly reflective areas can be restored to their detailed information.

On the basis of these subjective and objective evaluations, the all-in-focus imaging and lighting strategy with overexposure suppression effectively guides the developed imaging device to obtain highly detailed images with fully expressed global detail information for fault detection.

## 4. Conclusions

In this study, a cross-modal all-in-focus imaging method with overexposure suppression was proposed, which realizes its function by obtaining depth and pre-imaging information. Compared with existing methods, our method is unaffected by surface textures and ambient light, and it directly determines the focus required for the depth of the object plane, resulting in an all-in-focus imaging capability that ensures the large span depth imaging effects with efficient focusing. The low coupling device and method do not depend on the shape of the imaging surface, and the camera characteristics solve the problem of regional overexposures on highly reflective surfaces. Hence, drastic changes in the effects of the final overexposure suppression are avoided, showing good robustness.

Imaging experiments on non-Lambertian free-form surfaces were performed under no ambient light conditions, demonstrating that the new system adaptively obtains clear all-in-focus images without overexposure under all depth span and curved surface conditions inside non-Lambertian-shaped pipes. The system is small and demonstrates low coupling and self-adaptation. It is suitable for free-scene imaging in any irregular cavity requiring active lighting, pipes of different diameters, and highly reflective free-form surfaces.

Although the method of all-in-focus imaging with overexposure suppression in this paper can obtain the sequence images effectively, the fusion process of sequence images introduced in this paper needs three fusion algorithms, which are inefficient. In the future, further research will focus on studying more efficient special fusion algorithms to further improve the efficiency and robustness of image fusion.

## Figures and Tables

**Table 1:** Consistency parameter configuration.

Parameter	Resolution	Feedback Light Intensity	Aperture Value	ISO	Shutter Time		Saturation	Brightness	Sharpness
					Shutter Time of Pre-Imaging (ms)	Collection of Multiple Exposure Shutter Times (ms)			
Value	2592 × 1944	20 lux	2.8	800	50	{35, 50, 80, 120}	0	50	20

**Table 2:** Statistics of pre-imaging overexposures.

Light Source	T_light_	B_light_	L_light_	R_light_
Spot area (Size (IMG*_a_*))	590	3571	0	4713
Normalized spot area	0.0117	0.0711	0.0	0.0938

**Table 3:** Decision parameters of the dynamic lighting scheme.

Weight	k_O_	k_M_	kα	kβ
Value	0.7	0.3	30	5

**Table 4:** Process data statistics for lighting solutions under Viewpoint ii.

	T_light_-L_light_	T_light_-B_light_	T_light_-R_light_	L_light_-B_light_	L_light_-R_light_	B_light_-R_light_
S_or_	1580	4435	5770	4131	5790	6264
S_and_	0	54	165	206	0	2450
ORI	1	0.984	0.950	0.939	1	0.376
SOI	0.974	0.966	0.962	0.967	0.988	0.960
LC	0.992	0.979	0.953	0.947	0.961	0.551

**Table 5:** Results of depth segmentation and depth-of-field matching.

	<D*_j_*> (mm)	D_FM*j*_ (mm)
*j* = 1	219–292	250
*j* = 2	292–438	350
*j* = 3	438–878	584

**Table 6:** Process data statistics for lighting solutions under Viewpoint iii.

	T_light_-L_light_	T_light_-B_light_	T_light_-R_light_	L_light_-B_light_	L_light_-R_light_	B_light_-R_light_
S_or_	662	681	1920	491	1832	1802
S_and_	26	25	39	66	11	27
ORI	0.992	1	0.988	0.980	0.997	0.992
SOI	0.977	0.977	0.974	0.980	0.974	0.974
LC	0.988	0.993	0.984	0.979	0.990	0.987

**Table 7:** Objective evaluation results 1 of image clarity.

	IMG_FM1_	IMG_FM2_	IMG_FM3_	IMG_Fused_
V_EOG_	51.537	89.531	98.709	171.402
V_Tenengrad_ (10^3^)	1.294	2.342	2.879	4.377
V_Vollaths_ (10^3^)	0.591	0.610	0.607	0.839

**Table 8:** Objective evaluation results 2 of image clarity.

	SNR (db)	PSNR (db)
IMG_FM1_	14.6442	33.9384
IMG_FM2_	15.8984	35.1926
IMG_FM3_	17.5386	36.8323

## Data Availability

All data and code will be made available on request to the correspondent author’s email with appropriate justification.
